# Septic acute kidney injury: molecular mechanisms and the importance of stratification and targeting therapy

**DOI:** 10.1186/s13054-014-0501-5

**Published:** 2014-09-02

**Authors:** Eric D Morrell, John A Kellum, Núria M Pastor-Soler, Kenneth R Hallows

**Affiliations:** Renal-Electrolyte Division, Department of Medicine, University of Pittsburgh School of Medicine, Pittsburgh, PA 15261 USA; The Center for Critical Care Nephrology, University of Pittsburgh School of Medicine, Pittsburgh, PA 15261 USA; CRISMA (Clinical Research Systems Modeling of Acute Illness) Center, University of Pittsburgh School of Medicine, Pittsburgh, PA 15261 USA; Department of Critical Care Medicine, University of Pittsburgh School of Medicine, Pittsburgh, PA 15261 USA; Department of Cell Biology, University of Pittsburgh School of Medicine, Pittsburgh, PA 15261 USA

## Abstract

The most common cause of acute kidney injury (AKI) in hospitalized patients is sepsis. However, the molecular pathways and mechanisms that mediate septic AKI are not well defined. Experiments performed over the past 20 years suggest that there are profound differences in the pathogenesis between septic and ischemic AKI. Septic AKI often occurs independently of hypoperfusion, and is mediated by a concomitant pro- and anti-inflammatory state that is activated in response to various pathogen-associated molecular patterns, such as endotoxin, as well as damage-associated molecular patterns. These molecular patterns are recognized by Toll-like receptors (TLRs) found in the kidney, and effectuate downstream inflammatory pathways. Additionally, apoptosis has been proposed to play a role in the pathogenesis of septic AKI. However, targeted therapies designed to mitigate the above aspects of the inflammatory state, TLR-related pathways, and apoptosis have failed to show significant clinical benefit. This failure is likely due to the protean nature of septic AKI, whereby different patients present at different points along the immunologic spectrum. While one patient may benefit from targeted therapy at one end of the spectrum, another patient at the other end may be harmed by the same therapy. We propose that a next important step in septic AKI research will be to identify where patients lie on the immunologic spectrum in order to appropriately target therapies at the inflammatory cascade, TLRs, and possibly apoptosis.

## Introduction

Acute kidney injury (AKI) is a very common and especially formidable clinical problem in the ICU, where mortality rates approach 25% and soar to 50 to 60% when severe enough to require renal replacement therapy [1]. These statistics have not significantly improved over the past 50 years, though patients today are generally older and have more comorbidities [1]. The most common cause of AKI in hospitalized patients is sepsis [2], and AKI occurs with regularity even in non-severe sepsis where clinically evident hemodynamic changes are not readily apparent [3]. The combination of sepsis and AKI portends a dire clinical situation that is associated with a hospital mortality rate as high as 70% [4]. However, the basic pathophysiologic mechanisms undergirding the association between sepsis and the clinical manifestations of AKI are not completely understood.

In this review, we will first summarize the findings of published human studies that support the concept that the pathogenesis of septic AKI in humans should not be exclusively viewed in the context of distributive shock-associated ischemia, but rather also within the context of a dysregulated and ill-defined inflammatory response to septic stimuli. We then discuss why previous attempts at modulating the inflammatory response in septic AKI may have failed, and highlight more recent trials and therapies aimed at targeted treatment based on where patients lie along the immunologic spectrum. We also discuss the role Toll-like receptors (TLRs) have been shown to play in endotoxemic models of AKI, and again focus on why agents used to block TLR-related pathways may have failed to show significant clinical benefit. Finally, we review the data supporting and refuting a central role for apoptosis in septic AKI, and argue that much of the clinical manifestations in septic AKI occur prior to significant cell death secondary to apoptosis or necrosis.

### Septic acute kidney injury: is it hyperemic kidney injury?

The morphologic changes that are seen in classic ischemic and toxic acute tubular necrosis are often lacking [5,6] or are microscopically distinct in septic AKI [7,8]. While the use of renal biopsy in the management of septic AKI is exceedingly rare in many western nations, most human studies have found no consistent histopathologic changes in septic AKI, and acute tubular necrosis is infrequently observed [5,6]. In a systematic review of six studies describing histopathologic changes seen in septic AKI, Langenberg and colleagues [6] found that tubular epithelial cell necrosis was seen in only 22% of human samples taken after septic injury. A more recent study by Takasu and colleagues [8] examined post-mortem kidneys taken from 67 patients who died with sepsis, and while they found focal tubular injury in 78% of septic kidneys, the majority of tubular cells were normal.

Perhaps an even greater distinction between non-septic AKI and septic AKI might be made when examining the role of overall vascular hemodynamics. Traditionally, septic AKI has been thought to be secondary to a demand and perfusion mismatch, whereby renal blood flow was diminished in the midst of increased metabolic demand [9,10]. Furthering this idea are the FINNAKI and SEPSISPAM studies, two recently published trials that suggest septic patients with target mean arterial pressure higher than 65 mmHg might have less progression to AKI [11] and less need for renal-replacement therapy if they suffer from chronic hypertension [12].

Nevertheless, the data directly measuring overall kidney blood flow during sepsis suggest that flow is increased in the septic physiologic state. Despite a plethora of conflicting animal studies - some of which show overall increased kidney blood flow while others show overall decreased kidney blood flow [10,13] - Langenberg and associates [13] found only three human studies that have directly measured renal blood flow in septic AKI by invasive hemodynamic measurements. All three studies showed that septic AKI occurs in the midst of renal arterial vasodilation and preserved overall renal blood flow [14-16]. In another study with a small cohort of 10 patients with sepsis, renal blood flow was either preserved or increased [17]. Bellomo and associates [18] have thus proposed a new concept for understanding septic AKI hemodynamics, so-called hyperemic AKI.

Despite the above human studies that suggest an increase in overall renal blood flow in septic AKI, the complex interplay between regional microcirculation and hemodynamics may play a much more important role in overall kidney function during sepsis. As Langenberg and colleagues [13] and Molitoris [19] point out, glomerular filtration rate (GFR) and cellular perfusion can decrease even if overall renal blood flow is increased due to disproportionate vascular resistance between the afferent and efferent arterioles, regional microvascular flow rates, or renal venous congestion. There are limited human data regarding microcirculatory function in septic AKI. However, using implanted laser Doppler flow probes, Di Giantomasso and colleagues [20] showed that, in sheep hyperdynamic septic models using *Escherichia coli* infusion as a source of infection, regional redistribution of blood flow from the medulla to the cortex was absent during sepsis. Local inhibition of endothelial nitric oxide synthase by liposaccharide (LPS) may play a role in the decreased GFR observed in endotoxemic models of AKI, at least in rats [21]. In a hyperdynamic endotoxemic pig model, Cohen and associates [22] likewise observed a significant increase in overall kidney blood flow after LPS administration; however, almost all of the increased blood flow was shunted to the medulla, with no increase in cortical blood flow. In a sepsis pig model using peritonitis as a source of infection, Chvojka and colleagues [23] observed that while overall renal blood flow increased in septic pigs with AKI, renal perfusion pressure significantly decreased as a result of increased renal venous pressure. In parallel to this, there was a significant decrease in renal cortex microvascular perfusion as measured by laser Doppler blood flow, as well as increased renal venous acidosis and lactate concentrations [23]. These septic physiologic observations related to regional kidney blood flow have been found in humans as well. In a retrospective observational study, it was shown that elevated central venous pressure was associated with progression to AKI in septic patients (odds ratio = 1.22, 95% confidence interval (CI) 1.08 to 1.39), suggesting that venous congestion may contribute to septic AKI in humans [24].

An in-depth discussion about renal microcirculation in septic AKI is beyond the scope of this review. As mentioned above, there is a dearth of human data on this important topic and most of the animal data are conflicting and difficult to interpret, given the different models used and measurement modalities employed. While it is clear from above that ischemic and microvascular dysfunction may contribute to septic AKI, septic AKI can occur independently of overall renal hypoperfusion or ischemia [25]. Indeed, AKI occurs in up to one-third of non-critically ill patients with pneumonia, of which less than 10% received any vasopressor therapy [3]. Additionally, there have been many studies published over the past decade showing that plasma and urinary biomarkers are often distinct in septic and non-septic AKI (reviewed in [26]). This suggests a fundamental mechanistic distinction that may involve primarily a dysregulated immune response and possibly apoptosis as opposed to ischemic factors related to distributive shock [25].

### Sepsis-induced inflammation and its effects on the kidney

For many years, organ damage and other deleterious sequelae from sepsis were thought to result from a hyper-inflammatory ‘cytokine storm,’ triggered in response to either an infectious pathogen or major physiologic insult [27]. Thus, a significant amount of sepsis research in the 1990s focused on identifying specific cytokines that were associated with the stereotypical sepsis syndrome. However, while cytokines such as TNF-α and IL-1β were found in very high concentrations in septic patients, large clinical trials testing specific cytokine inhibition failed to show any clinical benefit [28,29].

As a consequence of these failed trails, new ideas regarding the pathogenesis and pathophysiology of sepsis emerged [30]. Utilizing pathologic data showing impaired immune function in some septic patients, Hotchkiss and Remick have both hypothesized that the host’s dysregulated and ill-defined inflammatory response to stimuli is associated with both pro- and anti-inflammatory states [31-33]. Many septic patients are chronically ill, and their development of sepsis is not necessarily associated with a hyper-inflammatory response [31]. One current goal in sepsis research is to identify where a septic patient lies on the immunologic spectrum, and identify which patients will benefit from specific therapies.

Indeed, the early trials focusing on inhibiting TNF-α or IL-1β did not risk-stratify their patients in terms of their individual inflammatory status or genetic predilections towards certain types of phenotypic responses [28,29]. It seems likely that the next step in sepsis and septic AKI treatment will be targeted therapy based on stratification of patients that have a higher likelihood of responding to various treatments. Panacek and colleagues first attempted this strategy with a randomized controlled trial to study afelimomab, an anti-TNF F(ab’)2 monoclonal antibody fragment, in septic patients with and without elevated serum levels of IL-6 [34]. In this study, for those patients who had serum IL-6 levels >1,000 pg/mL, they demonstrated a 4% absolute risk reduction in mortality at 28 days after treatment in patients treated with the agent than in those not treated. Although rates of kidney failure were not reported in this trial, there was a statistically significant improvement in the overall Sequential Organ Failure Assessment (SOFA) score starting at 48 hours after drug administration.

As an alternative to targeted treatment, some researchers have proposed implementing treatment strategies that are able to broadly regulate the overall inflammatory response in sepsis by using extra-corporeal blood purification. Multiple *in vitro* and rat *in vivo* studies have demonstrated that the broad reduction of inflammatory cytokines by hemoadsorption can protect against renal and liver injury [35], improve bacterial clearance [36] and mean arterial pressure [37], decrease apoptosis and tubular cell damage [38], and improve overall survival [35,37]. However, its efficacy in humans is still unknown. The Early Use of Polymyxin B Hemoperfusion in Abdominal Sepsis (EUPHAS) trial showed improved hemodynamics, organ function, and 28-day mortality in patients suffering from severe sepsis or septic shock from intra-abdominal Gram-negative infections who were treated with two sessions of polymyxin B hemoperfusion compared with controls [39]. Renal SOFA scores improved in the treatment group (-0.3, 95% CI -0.7 to 0.1 versus 0.6, 95% CI 0.1 to 1.1) versus control, as did overall cardiovascular function. However, there were several methodological flaws in this small trial, including a failure to measure endotoxin levels in the treatment and control groups and the use of 28-day mortality as a secondary endpoint. While these findings suggest the need for larger clinical trials to be performed before widespread implementation can be recommended, these preliminary results appear promising.

Although the *in vivo* animal studies cited above have shown that broad regulation of cytokine levels is possible with the use of cytokine-reducing resins, modulation of cytokine levels by continuous hemodialysis in humans has not been clearly demonstrated. Despite the very short half-life of many inflammatory cytokines, small initial studies suggested that high-volume hemofiltration might be able to modulate overall cytokine profiles in humans [40,41]. However, larger randomized controlled trials failed to show that continuous hemofiltration modulates cytokine levels [42], nor has any clinical benefit been demonstrated with the use of high-volume hemofiltration in the treatment of sepsis [42-44]. While the large VA/NIH Acute Renal Failure Trial Network study showed no mortality difference in the septic subgroup of patients who underwent more intense renal replacement, subsequent analysis has shown higher rates of non-recovery of kidney function and increased mortality in patients with higher circulating concentrations of pro- and anti-inflammatory cytokines and apoptosis markers [45]. This result again suggests that targeting certain subgroups along the immunologic spectrum might be beneficial not only in targeted cytokine therapy, but also in the broad modulation of inflammatory cytokines via hemofiltration and absorption.

Finally, the use of agents to augment immune function in patients suffering from sepsis-associated immunosuppression has shown clinical promise. As mentioned above, sepsis represents a complex inflammatory state with both pro- and anti-inflammatory characteristics. Thus, identifying and treating septic patients who are on the more immunosuppressed spectrum of sepsis with immune augmentation may improve outcomes. Meisel and associates [46] administered granulocyte-macrophage colony-stimulating factors (GM-CSFs) to septic patients who had decreased monocyte HLA-DR expression and found that monocyte immunocompetence could be restored in this subgroup of patients. While there was no change in the clinical outcomes that were measured (ventilator days and hospital length of stay), their data showed that biomarker-guided GM-CSF therapy in sepsis is safe and could hold promise in larger randomized controlled trials.

### Toll-like receptor-4 and the effects of sepsis on renal epithelial cells

Initial molecular research on sepsis suggested that the generalized inflammatory state described above is initiated when the innate immune system recognizes highly conserved pathogen-associated molecular patterns (PAMPs), such as LPS [47]. However, in the late 1990s Polly Matzinger [48] proposed the ‘danger model’, which argues that the immune system is more concerned with ‘danger signals’ than with foreign signals. Subsequent research has found that there are numerous ‘damage-associated molecular patterns’ (DAMPs) that can elicit immune responses similar to PAMPs [49]. The extent to which both PAMPs (a subgroup of DAMPs) and more broadly DAMPs activate the immune system and contribute to sepsis and septic AKI is not fully understood, but recent research has provided a significant amount of insight into the process.

**Figure 1:**
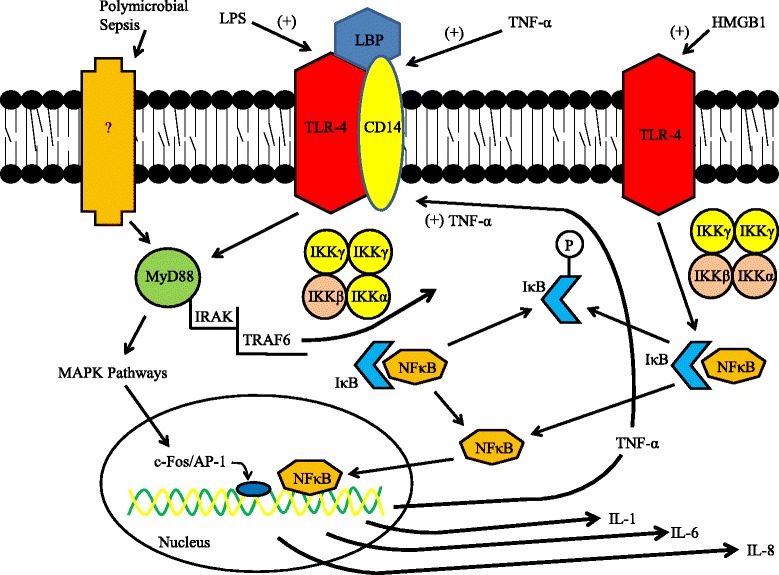
**Schematic illustration of the activation of the NF-κB transcription factor pathway by liposaccharide (LPS), TNF-α, high mobility group (HMG)B1, and polymicrobial sepsis.** LPS binding to Toll-like receptor-4 (TLR-4) is dependent on its interaction with circulating LPS-binding protein (LBP) and the subsequent interplay of this complex to membrane bound CD14 [90]. This LPS-LBP-CD14-TLR-4 complex is thus anchored to various cells within the kidney (especially within the apical brush border of proximal tubules) [60], where it initiates intracellular signaling cascades. CD14 gene expression is upregulated by LPS [91] in human kidney proximal tubular epithelial cells and by TNF-α [92] in mouse renal interstitial and tubular epithelial cells. Activated TLR-4 via LPS stimulates the MyD88 pathway [47,93,94], and ultimately induces phosphorylation of IκB by IκB-kinase-β (IKKβ; tan-colored) within the IKK complex [65]. Phosphorylated IκB is then released from NF-κB, allowing NF-κB to migrate to the nucleus to modulate transcription of various cytokines as shown. HMGB1 activation of TLR-4 activates NF-κB via an alternative mechanism whereby both IKKα and IKKβ (tan-colored) are implicated [54,55]. In cecal ligation and puncture models of sepsis, MyD88 has been shown to be activated independent of TLR-4 by unknown upstream cell surface receptors [72,75]. IRAK, interleukin receptor-associated kinase; MAPK, p38 mitogen-activated protein kinase; TRAF, TNF receptor associated factor.

TLRs have provided a link between host and pathogen [50,51], as well as host and DAMPs [52]. In the 1990s, TLR-4 was found to be the main receptor that binds LPS, leading to the release of TNF-α and IL-1 and activation of numerous downstream intracellular signaling pathways via the NF-κB transcription factor [50,53] (Figure 1). Additionally, in sterile models of inflammation, TLR-4 has been shown to respond to DAMPs, such as high mobility group-B1 [54-56]. Indeed, the capacity of TLRs to respond to PAMPs as well as other DAMPs provides a potential link between systemic inflammation and kidney damage that is independent of ischemia and hypotension. In the kidney, TLR-4 is constitutively expressed in mouse primary renal tubular epithelial cells [57] and in mouse cells harvested from *in vivo* experiments [58], in renal tubular cells harvested from rats [59,60], and in human kidney tubular epithelial cells taken from surgical patients [61]. TLR-4 expression in the glomeruli is controversial [59,60], but its expression in tubular epithelial cells has been shown to modulate septic AKI and inflammation (Figure 1) in multiple models of injury [60,62,63]. The final downstream mechanisms that produce AKI after TLR-4 activation are complex and incompletely understood. Of note, recent work suggests that one important mechanism underpinning septic AKI is that this inflammatory pathway induces renal tubular transport dysfunction, which enhances NaCl delivery to the macula densa and increases tubuloglomerular feedback, thus decreasing GFR [64].

NF-κB is a highly expressed transcription factor that is often activated in response to cellular stresses and induces the synthesis and release of TNF-α, IL-1, IL-6, and IL-8 [65]. NF-κB-mediated processes occur in isolated primary rat proximal tubular epithelial cells [66], but the overall contribution of inflammatory cytokine levels in the kidney from local versus systemic production of those cytokines is unknown. Experiments performed on TLR-4 knockout mice transplanted with TLR-4 expressing kidneys suggest systemic production may be more important [62]. Direct inhibition of NF-κB with agents such as methyl-2-acetamidoacrylate (an ethyl pyruvate analogue) [67], ethyl pyruvate [68], and nicotinic acetylcholine [69] can lower cytokine levels in mice (TNF-α, IL-6, IFN-γ, and IL-10) and have been associated with improved clinical characteristics in mice suffering from septic AKI [67-69].

However, a recent randomized controlled trial examining the effect of an MD2-TLR-4 antagonist failed to show any mortality benefit in human patients suffering from severe sepsis [70]. In this study, none of the patients enrolled were stratified in terms of what type of infection they were suffering from (polymicrobial, Gram-positive, or Gram-negative). Moreover, levels of endotoxin were not measured, which leaves open the possibility that some patients may have benefited depending on where they stood on the immunologic spectrum. Nonetheless, this trial possibly highlights some of the pitfalls of associating LPS endotoxemia with human sepsis that have manifested themselves over the past 20 years in sepsis research.

Indeed, multiple studies have shown that in polymicrobial and cecal ligation and puncture models the mechanism of damage induced by sepsis may be very different than in LPS-induced models [71], might be independent of TLRs altogether [72-75], and instead could be mediated by direct MyD88 regulation (Figure 1) [72,75]. Interestingly, the presence of a specific TLR-4 mutation (D299G) in humans, which impairs LPS signaling, has not been shown to influence TNF-α, IL-6, and IL-10 levels in sepsis [73]. Nor has this particular allele been associated with any change in outcome or development of sepsis in patients undergoing major visceral surgery [73] or in patients with meningococcal disease [76]. The effect of this specific mutation has not been examined in kidneys directly, and its study may provide significant insight into the mechanisms of septic AKI in humans. Of note, the use of various mouse models to study human inflammation and sepsis has recently been questioned due to the significant differences in the two species’ genomic responses to stress [77].

### Does apoptosis have a role in septic acute kidney injury?

**Figure 2:**
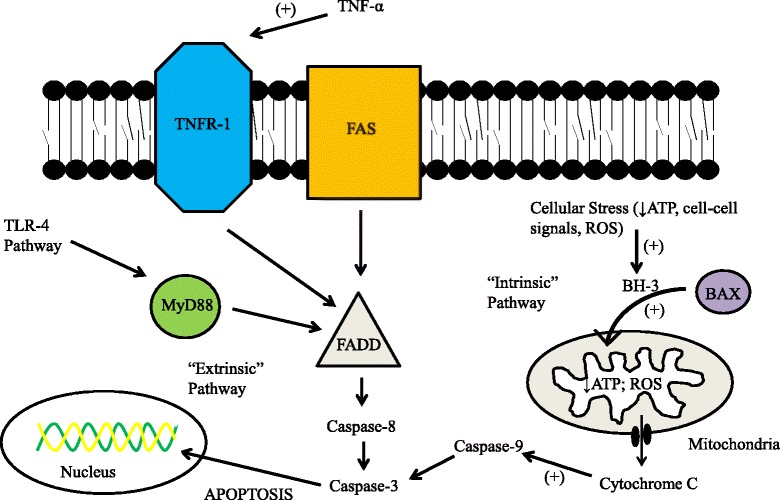
**Schematic illustration of the molecular processes leading to apoptosis in renal proximal tubular epithelial cells.** So-called death receptors such as Fas, TNF receptor-1 (TNFR-1), or the TNF-related apoptosis-inducing ligand (TRAIL) receptors bind with extracellular ligands to activate downstream activator caspases [78]. This process is usually mediated by the FADD (Fas-associated protein with death domain) receptor, and ultimately activates caspase-3 - the effector molecule. Within the cell, the intrinsic pathway is initiated via cellular stresses: reactive oxygen species (ROS), nitric oxide, decreased ATP, or cell-cell/cell-matrix perturbations [95]. In response to those stresses, BAX migrates into the mitochondria via activation from BH3-only proteins within the Bcl-2 family [96]. Once they have entered the mitochondria, BAX and other pro-apoptotic proteins form pores within the mitochondrial wall, allowing for the release of cytochrome c, which triggers caspase-dependent and -independent pathways [95].

The traditional model of apoptosis in kidney and other cells is similar [78]. Processes involving the mitochondria and cell respiration are labeled ‘intrinsic,’ while binding of cell surface proteins leading to subsequent intracellular signaling events are termed ‘extrinsic’ processes (Figure 2) [78]. However, the mechanisms whereby cells in the septic kidney succumb (or do not succumb) to apoptosis are still under investigation. One important route may be via the tumor necrosis factor receptor-1 (TNFR-1; Figure 2). TNFR-1 knockout mice were resistant to LPS-induced kidney failure while they also manifested much less apoptosis and neutrophil accumulation, suggesting a direct role for both necrosis and apoptosis in septic AKI [79]. Downstream of TNFR-1, Jo and colleagues [80] have shown that apoptosis in LPS, TNF-α, and IL-1 exposed HK-2 cells is likely dependent in part on Fas and caspase expression. Caspase inhibition has also been shown to prevent apoptotic proximal, distal, and peri-tubular cell death in LPS models of septic AKI [81]. Cantaluppi and associates [38] have demonstrated a role for the Fas and caspase-mediated pathway in septic AKI as well. Using primary human proximal tubule epithelial cells exposed to septic human plasma, they showed that: 1) apoptosis as detected through DNA TUNEL assays was significantly increased in cells exposed to septic plasma; 2) levels of Fas and caspase-3, -8, and -9 were all significantly increased in response to septic plasma; and 3) levels of those effector signals and apoptosis can be decreased by removal of inflammatory mediators via a resin adsorption method to filter septic plasma [38]. Indeed, initiation of apoptosis was observed in cultured kidney tubular cells exposed to plasma taken from patients suffering from severe burns and septic AKI [82].

However, in most of the *in vitro* and *in vivo* studies examining the presence or role of apoptosis in septic AKI, there were major methodological shortcomings. LPS was used as a model for sepsis in most of the studies cited above [79-81], as well as many experiments evaluating kidney tubular apoptosis in primary mouse distal convoluted tubule cells [83] and mouse MCT proximal tubule cells [84]. As mentioned in the previous section, LPS endotoxemia is not a model of sepsis. Perhaps even more confounding to the argument that apoptosis is a major factor in septic AKI is the lack of histological evidence in patients with septic AKI. Histologic sections of septic human kidneys generally demonstrate very little necrosis or cell damage [5-8]. Moreover, a significant amount of data taken from ‘warm’ autopsies [5,8] has also failed to show substantial apoptosis, at least as measured by conventional light microscopy [5], electron microscopy [8], and DNA agarose gel electrophoresis for apoptotic laddering [5]. In the seminal septic autopsy study performed by Hotchkiss and colleagues [5], they failed to observe any evidence of kidney apoptosis despite the fact that 65% of their samples were taken from patients with clinically manifest sepsis-associated kidney dysfunction. While apoptosis likely plays a major role in lymphocytes and other cells within the human body during sepsis [30], its role in septic AKI is unclear. Further study using more physiologically relevant models of sepsis (as opposed to LPS) are needed to help reconcile much of the conflicting data that currently exist.

Perhaps one of the reasons for the discrepancy between histologic data and functional performance in septic AKI is that most of the insults occur prior to significant cell death from apoptosis and necrosis. Indeed, apoptosis and necrosis are end-stage manifestations of cellular insults, and significant impairment of cellular function can occur before the histologic and genomic changes that are measured in most apoptosis and necrosis assays. Despite a measured decrease in kidney perfusion in their septic mouse model, Tran and associates found no elevation in hypoxia-inducible factor-1α, a transcription factor that is significantly upregulated [85] in hypoxic and ischemic rat kidneys [86]. They hypothesized that this was due to significantly reduced oxygen consumption by kidney proximal tubule cells during sepsis [86]. Further study showed that this process was likely mediated by PPARγ coactivator-1α (PGC-1α), a major regulator of mitochondrial function. PGC-1α was significantly downregulated in septic rats exposed to LPS and TNF-α, whereas increased levels of PGC-1α were associated with restored oxygen consumption and improved kidney function [86]. While increased expression of hypoxia-inducible factor-1α was not seen in mice exposed to LPS [86], sepsis has been associated with significant intra-renal oxidative and nitrosative stress that is mediated by inflammatory cytokines rather than local hypoxia or ischemia [87,88]. The idea of ‘cellular stunning’, whereby a cell that is living within a septic environment is trying to defend itself from death by decreasing its metabolic activity, has been proposed as a mechanism not only in sepsis by Hotchkiss and Karl [30], but also in ischemic models in the heart [89].

## Conclusion

While the relative contributions that ischemia, TLR-4-mediated pathways, apoptosis, and other subcellular processes have towards overall organ dysfunction are currently unclear, the abundance of research in the area has shed a significant amount of light on the basic mechanisms that drive septic AKI. Perhaps the most important finding is that therapeutic approaches targeted at a single mediator for all patients may not succeed given the protean nature of the disease. Indeed, targeted inhibition of cytokines such as TNF-α and IL-1 as well as TLR antagonists have failed to show consistent and significant clinical benefit, most likely because only certain subsets of septic patients fit an immunologic profile that would benefit from those therapies. An important next step in sepsis research should be to identify immunologic markers in patients that can help tailor specific therapies for patients based on where they lie along the immunologic spectrum.

## Abbreviations

AKI: Acute kidney injury
CI: Confidence interval
DAMP: Damage-associated molecular pattern
GFR: Glomerular filtration rate
GM-CSF: Granulocyte-macrophage colony-stimulating factor
IFN: Interferon
IL: Interleukin
LPS: Lipopolysaccharide
NF: Nuclear factor
PAMP: Pathogen-associated molecular pattern
PGC-1α: PPARγ coactivator-1α
SOFA: Sequential Organ Failure Assessment
TLR: Toll-like receptor
TNF: Tumor necrosis factor
TNFR: Tumor necrosis factor receptor
